# Transient receptor potential canonical type 6 (TRPC6) O-GlcNAcylation at Threonine-221 plays potent role in channel regulation

**DOI:** 10.1016/j.isci.2023.106294

**Published:** 2023-02-28

**Authors:** Sumita Mishra, Junfeng Ma, Desirae McKoy, Masayuki Sasaki, Federica Farinelli, Richard C. Page, Mark J. Ranek, Natasha Zachara, David A. Kass

**Affiliations:** 1 Division of Cardiology, Department of Medicine, Johns Hopkins University School of Medicine, Baltimore, MD, USA; 2 Department of Oncology, Lombardi Comprehensive Cancer Center, Georgetown University Medical Center, Washington, DC, USA; 3 Department of Chemistry and Biochemistry, Miami University, Oxford, OH, USA; 4 Department of Biological Chemistry, Department of Oncology, Johns Hopkins University, Baltimore, MD, USA; 5 Department of Pharmacology and Molecular Sciences, Johns Hopkins University, Baltimore, MD, USA

**Keywords:** Biochemistry, Cellular physiology, Cell biology

## Abstract

Transient receptor potential canonical type 6 (TRPC6) is a non-voltage-gated channel that principally conducts calcium. Elevated channel activation contributes to fibrosis, hypertrophy, and proteinuria, often coupled to stimulation of nuclear factor of activated T-cells (NFAT). TRPC6 is post-translationally regulated, but a role for O-linked β-N-acetyl glucosamine (O-GlcNAcylation) as elevated by diabetes, is unknown. Here we show TRPC6 is constitutively O-GlcNAcylated at Ser14, Thr70, and Thr221 in the N-terminus ankryn-4 (AR4) and linker (LH1) domains. Mutagenesis to alanine reveals T221 as a critical controller of resting TRPC6 conductance, and associated NFAT activity and pro-hypertrophic signaling. T→A mutations at sites homologous in closely related TRPC3 and TRPC7 also increases their activity. Molecular modeling predicts interactions between Thr221-*O*-GlcNAc and Ser199, Glu200, and Glu246, and combined alanine substitutions of the latter similarly elevates resting NFAT activity. Thus, O-GlcNAcylated T221 and interactions with coordinating residues is required for normal TRPC6 channel conductance and NFAT activation.

## Highlights

• TRPC6 is constitutively O-GlcNAcylated at T221 to suppress basal conductance
• A T221A mutant TRPC6 has much greater conductance and NFAT-activation
• Reducing but not raising O-GlcNAc alters T221-dependent TRPC6 function
• T221 coordinates with S199, E200, and E246 to control basal channel activity

## Graphical abstract

**Figure:**
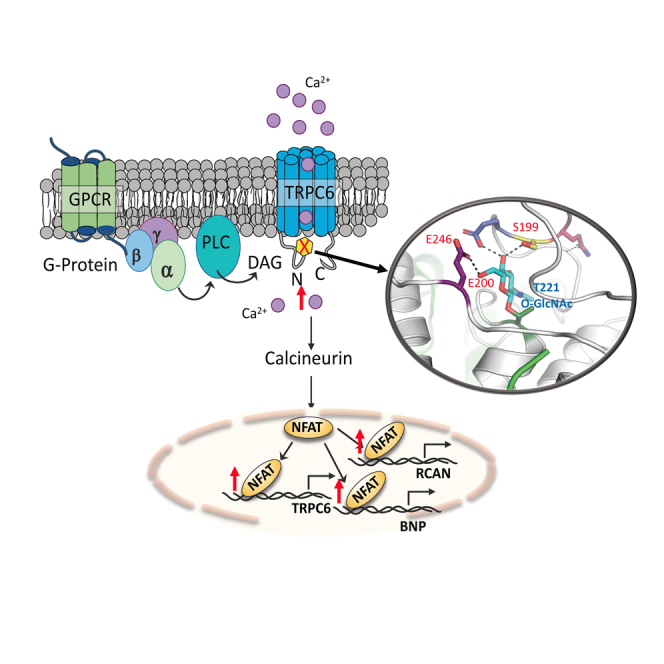

## Introduction

Transient receptor potential canonical type-6 (TRPC6) is a non-voltage gated transmembrane cation channel conducting primarily Ca^2+^.^1^ It is expressed at low basal levels in many cell types, and its upregulation and/or post-translational activation is thought to contribute to the pathophysiology of many diseases. They include glomerulosclerosis with podocyte dysfunction,^2^ pathological cardiac hypertrophy,^3^^,^^4^ dystrophin deficient muscular dystrophy,^5^^,^^6^ wound and pathological fibrosis,^7^ pulmonary vascular hypertension,^8^^,^^9^^,^^10^ wound healing^7^^,^^11^ and autoimmune disease.^12^ This pleiotropic behavior has led to growing interest in TRPC6 as a pharmacological target, and selective oral inhibitors have been developed^13^ and are in human trials for renal disease.

TRPC6 is primarily stimulated by G-protein coupled receptor signaling via diacylglycerol and phospholipase C,^1^ and by mechanical stretch.^5^ Calcium conducted by TRPC6 stimulates calcium-calmodulin activated phosphatase calcineurin (CaN) to dephosphorylate nuclear factor of activated T-cells (NFAT). This results in NFAT nuclear translocation and altered gene expression.^14^^,^^15^ NFAT consensus sequences within the TRPC6 promoter provide a positive feedback loop that amplifies TRPC6 associated signaling. In humans, TRPC6 gain of function (GOF) mutations result in familial segmental glomerulosclerosis.^16^^,^^17^^,^^18^ GOF mutations reside in both cytoplasmic N-terminus ankyrin repeat and C-terminus domains that are physically fairly close,^19^^,^^20^ and can function by disrupting inhibitory calcium binding sites.^21^

TRPC6 conductance and its associated NFAT activation are normally controlled by post-translational modifications.^22^^,^^23^^,^^24^^,^^25^ For example, oxidant stress coupled to NADPH oxidases NOX2 and NOX4 stimulates TRPC6 signaling.^26^^,^^27^N-glycosylation at N472 and N561^28^ and phosphorylation at S14^23^ are required for normal TRPC6 membrane localization and activity. Phosphorylation can both increase (at T487 by calcium-calmodulin activated kinase, CaMKII),^29^ or decrease (at T70 and S262 by cGMP-activated kinase, cGK1α)^30^^,^^31^ channel conductance and associated TRPC6 signaling. Hyperglycemia is also associated with TRPC6 and NFAT upregulation in kidney,^32^^,^^33^^,^^34^ monocytes,^35^^,^^36^ and platelets.^37^ This latter observation first led us to speculate TRPC6 may also be modified by O-linked β-N-acetyl glucosamine (O-GlcNAc).^38^^,^^39^^,^^40^O-GlcNAc-ylation occurs on serine or threonine residues^41^^,^^42^ and can alter protein activity, protein-protein interactions, structure, subcellular localization, and stability.^43^^,^^44^^,^^45^ Two recent comprehensive databases, the O-GlcNAc protein database (www.oglcnac.mcw.edu) and the O-GlcNAc Atlas,^46^ do not presently report TRPC6 or closely associated TRPC3 and TRPC7 as being O-GlcNAc-modified.

Here, we tested whether TRPC6 is modified by O-GlcNAcylation, and if so where this occurs and what its targeted residues impact. Proteomics identified three TRPC6 residues in the N-terminus to be constitutively O-GlcNAcylated, of which T221 conferred potent basal suppression of channel conductance and corresponding NFAT activity. Threonine 221 resides in the fourth ankyrin repeat domain, and modeling analysis found its O-GlcNAcylation enhances coordination with neighboring residues at Ser199, Glu200, and Glu246 that contribute to the constraint of basal TRPC6 conductance and NFAT signaling. The findings identify a new critical regulatory region of the protein that may help future design of pharmacological modulators.

## Results

### TRPC6 is constitutively O-GlcNAcylated in the N-terminus cytoplasmic domain

**Figure 1:**
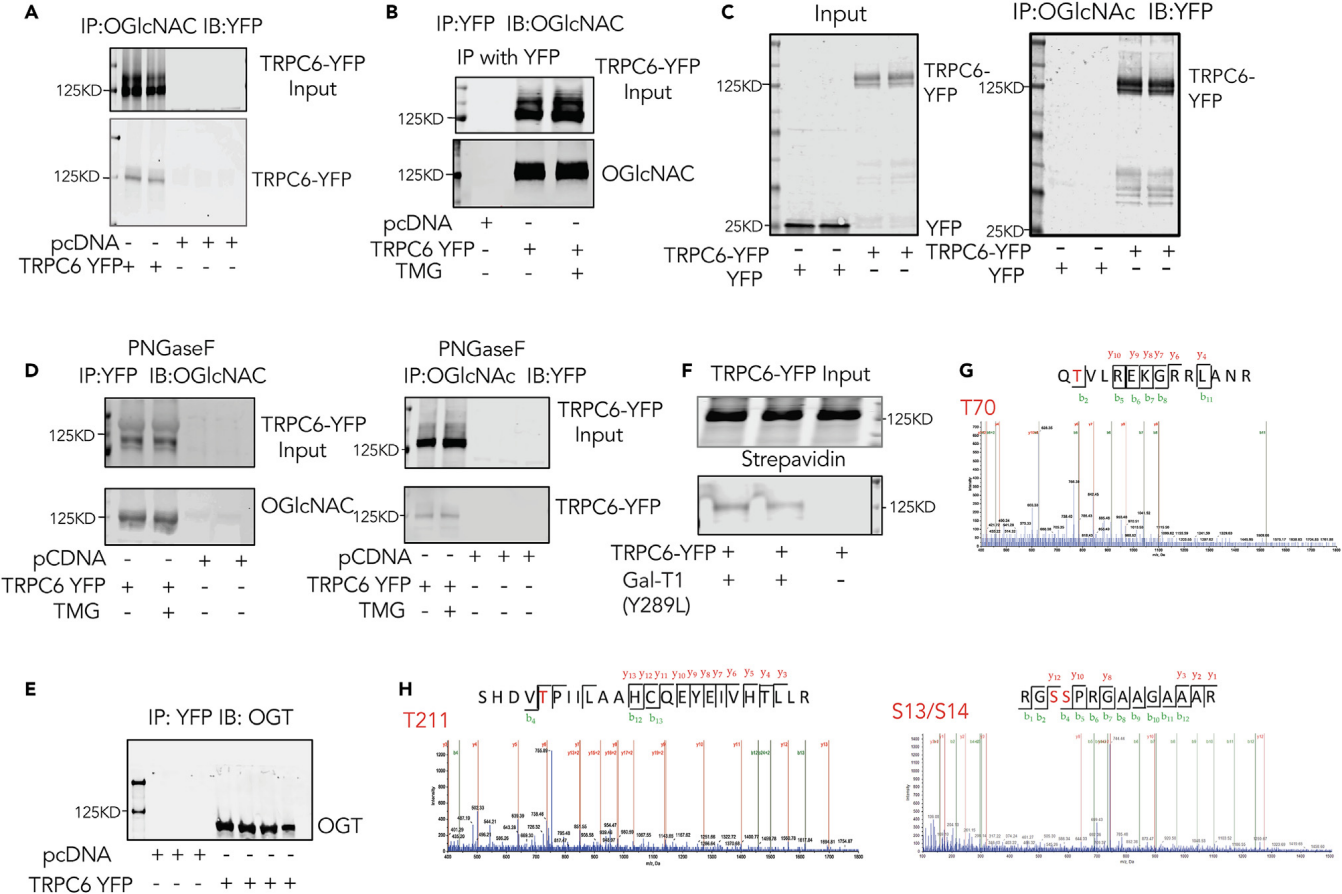
TRPC6 is constitutively OGlcNAcylated (A)HEK-293T expressing TRPC6-YFP or pcDNA were subjected to immunoprecipitation (IP) using O-GlcNAc antibody and then immunoblotted (IB) using GFP antibody (detects YFP-labeled TRPC6). Upper gel shows input, lower IP. (B) Same experiment but using YFP for the IP, and O-GlcNAc for IB. In this study, a group of cells were also exposed to 10 μM Thiamet G (TMG) for 24 h to assess if enhancing O-GlcNAcylation altered the signal associated with TRPC6. (C) Similar experiment using YFP as the control. The input shows either YFP or TRPC6-YFP expressed. After IP for OGlcNAc, IB for YFP shows only in the TRPC6-YFP band region. (D) IP blots with cells pre-treated with 0.2 μg of N-glycan-specific endoglycosidases PNGase F, showing persistence of TRPC6 O-GlcNAcylation. pcDNA serves as the control. (E) HEK-293T cells were co-transfected with TRPC6-YFP and OGT-Flag, and YFP IP performed and probed with OGT Ab. The data shows co-IP of both TRPC6-YFP and OGT-Flag. (F) O-GlcNAc metabolic labeling of TRPC6-YFP by GlcNAz and mutated Gal-T1 to enable click chemistry that adds biotin to O-GlcNAcylated residues. The upper band shows streptavidin beads isolation lysate probed for TRPC6-YFP showing equal input (upper band). For the lower band, GFP-Ab (detecting YFP) was used for the IP, and then probed with streptavidin IRDye to assess for biotinylation. Only TRPC6 subject to the click-chemistry biotinylation based on its having O-GlcNAcylation was detected. (G and H) Results of mass spectroscopy analysis of TRPC6-YFP O-GlcNAcylation revealed three peptides G*S*^*14*^SPRGAAGAAAR,Q^*70*^*T*VLREKGRRLANR,SHDV^*221*^*T*PIILAAHCQEYEIVHTLLR. The position of O-GlcNAc sites are highlighted.

To examine if TRPC6 is O-GlcNAcylated, HEK-293T cells were transfected with plasmid expressing a TRPC6-YFP fusion protein to facilitate detection of expression and provide a robust epitope for immunoprecipitation (IP). IP was performed using either YFP or O-GlcNAc as bait, and the precipitate then probed with anti-GFP (works as anti-YFP) or monoclonal anti-*O*-GlcNAc antibodies. The pull-down revealed that recombinant TRPC6 (∼130 kD) is constitutively O-GlcNAcylated (Figures 1A and 1B). Here the control was pcDNA. To confirm YFP itself was not the O-GlcNAc target, we repeated the experiment in cells expressing YFP alone or TRPC6-YFP. Only TRPC6-YFP was found in the oGlcNAc IP lysate (Figure 1C).

TRPC6 is known to be constitutively N-glycosylated, and to confirm that this was not influencing the O-GlcNAc signal, we repeated the experiment using lysates pre-incubated with PNGaseF to remove this modification. The molecular weight was lower and double-band appearance was less after PNGaseF treatment as expected^47^ (Figure S1). However, the TRPC6 protein was still bound by antibodies recognizing O-GlcNAc (Figure 1D). To test if the basal levels of TRPC6 O-GlcNAcylation could be augmented by stimulating OGT, we treated cells with TMG (Figure 1B) but found no change in the O-GlcNAc band intensity. Additional support for the targeting of TRPC6 by O-GlcNAcylation is provided by finding co-immunoprecipitation of O-GlcNAc transferase (OGT), the enzyme that generates this modification, with YFP in cells expressing TRPC6-YFP (Figure 1E). Lastly, we performed a Click-it^TM^ assay in HEK-293T cells, in which O-GlcNAcylated protein residues were first selectively labeled by tetra-acetylated azide-modified *N*-acetylglucosamine (GlcNAz) by Y289L-b4-Gal-T1, and then co-tagged with biotin alkyne by click chemistry. The presence of biotinylation was confirmed by streptavidin-based immunoblot (upper gel), but TRPC6-YFP was only detected in cells treated with Y289L-b4-Gal-T1, further supporting TRPC6 as constitutively O-GlcNAcylated (Figure 1F).

To directly identify O-GlcNacylation of TRPC6 and the residues involved, we performed tandem mass spectrometry. Cell lysates from HEK-293T cells over-expressing TRPC6-YFP were subjected to pull-down using anti-YFP antibody magnetic beads. The immunoprecipitant was subjected to in-gel digestion, and the digests analyzed by nanoUPLC-MS/MS analysis. Three O-GlcNAc-containing peptides in TRPC6 were identified and the modified residues mapped to Ser13/14, Thr70 and Thr221 (Figures 1G and 1H). Of these, T221 had not been previously reported as being modified by any PTM, whereas T70 was a known phosphorylation target of cGK1α^30^ and S14 by cdk5c (Liu et al., 2020).^25^ T221 in particular resides in the AR4 domain of TRPC6 and is highly conserved across species (Figure S2).

### T221 O-GlcNAcylation is a primary regulator of basal TRPC6 function

**Figure 2:**
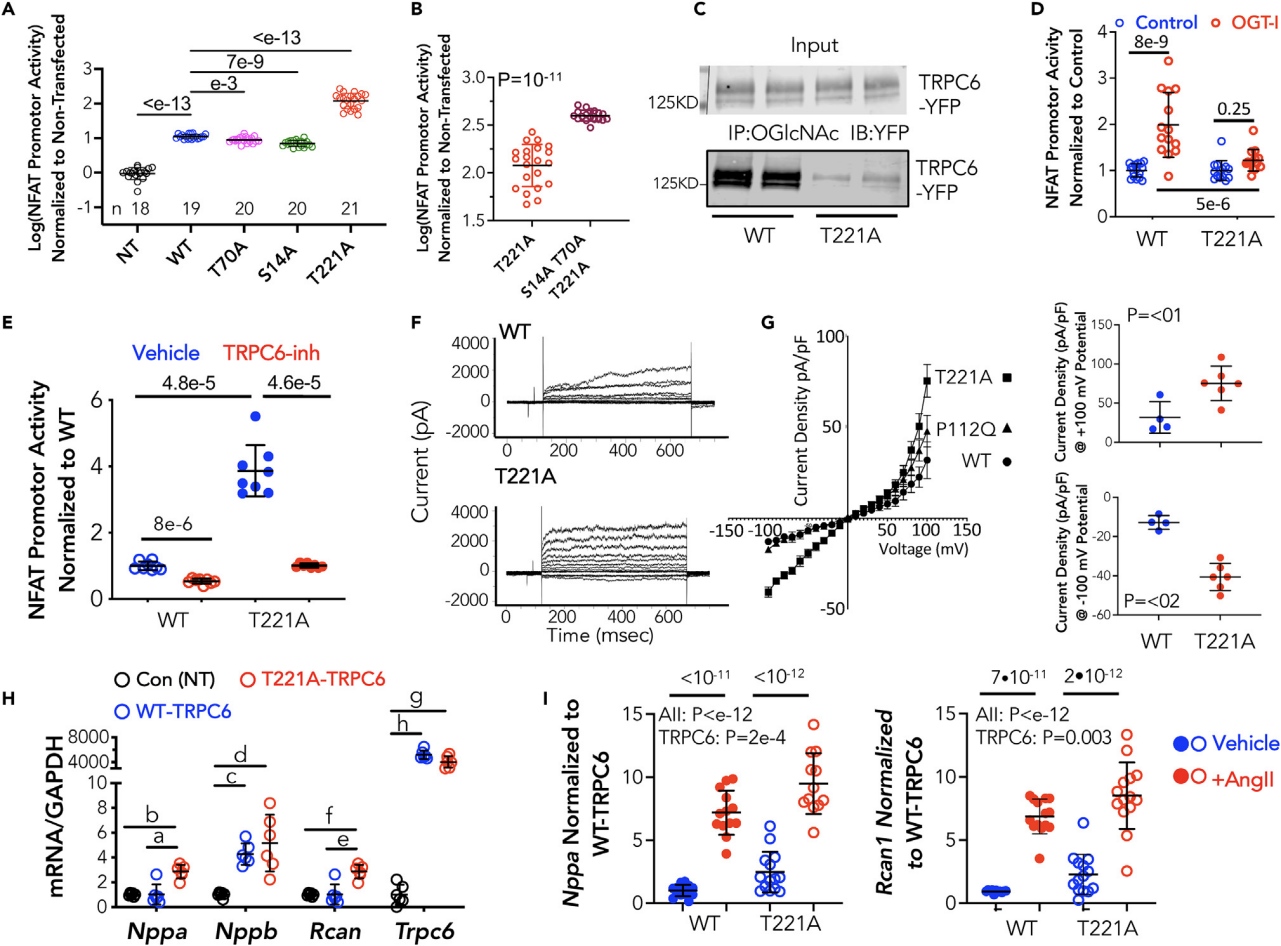
Substitution of T221 with T221A results in hyperactive TRPC6 In all panels showing group data, mean ± SD is displayed for each group. (A) NFAT promoter activity in cells transfected with pcDNA (C), TRPC6-YFP (WT) or TRPC6-YFP mutants (S14A, T70A, T221A) for 24 h (n/group provided in figure). Data are log-transformed, analysis by Brown-Forsythe Welch ANOVA, p-values from Dunnett’s multiple comparison test (MCT). (B) Co-expression of S14A, T70A, and T221A (n = 18) versus T221A alone (n = 21). p-value Mann-Whitney test. (C) IP assay in HEK293 cells expressing WT or T221A mutant TRPC6, with IP using OGlcNAc Ab, and the probed for YFP. Cells with WT TRPC6 show O-GlcNAcylated TRPC6, whereas the signal is faint in the T221A mutant. (D) NFAT activity assay in HEK cells expressing WT or T221A TRPC6 in the presence of OGT inhibition (OGT-I) or vehicle (Control). Data are shown normalized to the control in each respective TRPC6 genotype group. Analysis by 2W-ANOVA, Sidak’s multiple comparisons test, (n = 13–16/group). (E) NFAT promoter activity because of expression of WT or T221A TRPC6 is markedly blunted by selective TRPC6 antagonist, BI 749327 (n = 8/group, Welch ANOVA, p-values Dunnett’s MCT.). (F) Example current-time-voltage tracings in HEK-293 cells expressing WT or T221A mutant TRPC6. (G) Summary current density versus voltage relations for WT, T221A, and P112Q TRPC6. Current density comparisons for WT and T221A at a transmembrane voltage of +/− 100 mV are shown to the right, p value Mann-Whitney test. The current density plot also shows a gain-of-function mutation P112Q falls in between WT and T221A at positive voltages but has no impact on current density at negative voltages. (H) Gene expression of hypertrophic response genes in cardiomyocytes expressing control (GFP), and WT or T221A mutant TRPC6 adenovirus for 48h. Genes are: A-type and B-type natriuretic peptide (*Nppa, Nppb),* regulator of calcineurin (*Rcan1*) and TRPC6 (*Trpc6*), each normalized to *Gapdh.* p-values from Dunnett’s MCT following Welch ANOVA; a: 6e-4, b: 8e-5, c:4e-5, d: 0.005, e: 6e-4, f: 8e-5, g:3e-4, h:2e-5. (n = 6 for *Trpc6*, n = 7 for the rest). (I) Hypertrophic gene expression in cardiomyocytes exposed to angiotensin II versus vehicle control, and expressing either WT or T221A TRPC6. AII augments NFAT promoter activity independent of TRPC6 genotype with the genetic mutation increasing activity overall. Results of 2WANOVA, Sidak’s MCT, n = 12–14/group. AII is p-value for effect of angiotensin II, TRPC6 the effect of the mutation. Interaction was >0.36 (NS) for both genes.

To investigate the functional roles of the three O-GlcNAcylated residues in TRPC6, we performed site-directed mutagenesis substituting an alanine at each site to prevent the PTM. Compared to wild-type (WT) TRPC6, S14A and T70A mutants modestly lowered basal NFAT activity by −19 and −36%, respectively (both p < 0.002). In contrast, the T221A mutation displayed markedly increased promoter activity (11-fold over WT, p < 10^−13^, Figure 2A). When all three mutations were introduced, NFAT activity rose further than with T221A alone (Figure 2B). This identifies T221 as the dominant regulating site and shows influences from the other two sites depends on T221 status.

The prominent impact of the T221A mutation could potentially be due to intrinsic changes in the channel function from the mutation independent of its blocking O-GlcNAcylation. To address this, we transfected cells with WT or T221A TRPC6-YFP, performed IP using O-GlcNAc-Ab, and then probed the precipitate for TRPC6-YFP. As before, we observed a signal when the WT channel was expressed but little with the T221A mutant (Figure 2C). In a second experiment, similar cells were incubated with either the OGT-inhibitor (OSMI-1) to reduce O-GlcNAcylation, or vehicle control, and NFAT promoter activity measured (Figure 2D). Here, data are displayed normalized to control for each TRPC6 genotype to facilitate their comparison. In WT-TRPC6 expressing cells, OGT inhibition enhanced NFAT promoter activity, whereas this was blocked in cells expressing T221A-TRPC6 (p = 0.0004 for difference in OGT-inhibitor effect between genotypes by two-way ANOVA).

We next tested whether the T221A mutation disrupted TRPC6 membrane expression or its external structure, using a potent selective TRPC6 antagonist (BI 749327)^13^ and found this inhibitor significantly reduced NFAT promoter activity in both WT TRPC6 and T221A mutant (Figure 2E). This indicates TRPC6-T221A membrane localization and external structure were likely intact. By contrast, N-glycosylation is required for normal membrane localization.^28^

To determine the functional impact of T221A mutagenesis, TRPC6 calcium conductance was measured by patch-clamp assay in HEK-293T expressing WT or T221A TRPC6. Figure 2F shows typical raw current-voltage-time tracings, and Figure 2G the summary data. Bidirectional voltage-dependent current increased by 75–80% in cells expressing T221A-TRPC6 versus WT. This is similar to changes in current induced by angiotensin stimulation of TRPC6 conductance.^30^ Here we also compared the changes in current density-voltage dependence with T221A to a well described GOF mutation P112Q associated with aggressive renal disease.^16^ At positive voltages (outward current), the P122Q mutation fell intermediate between WT and T221A, whereas at negative voltages (inward current), P122Q had no impact, whereas this was still markedly increased by T221A (Figure 2G). These data identify T221 as a bidirectional regulator of basal TRPC6 Ca^2+^ current.

Lastly, we examined the impact of T221 on resting and agonist stimulated NFAT signaling in cardiomyocytes that normally express low levels of TRPC6 but on its activation display pathological hypertrophic signaling.^30^ Myocytes were transfected with either WT or T221A TRPC6 achieving a high relative level of *Trpc6* expression mostly reflecting the very low baseline. We observed increases in two prominent biomarkers of hypertrophic signaling, *Nppa* (A-type natriuretic peptide) and *Rcan1* (regulator of calcineurin-1) in cells expressing the T221A mutant, whereas *Nppb* (B-type natriuretic peptide) rose similarly with both (Figure 2H). We also tested if expression of T221A in myocytes limits or amplifies enhancement of NFAT signaling induced by angiotensin II stimulation. As before, myocytes expressing T221A TRPC6 exhibited increased expression of both *Nppa* and *Rcan1,* but AII stimulation still augmented this further and similarly as in cells with WT TRPC6 (Figure 2I). There was no no significant interaction (2W-ANOVA) between AII or genotype effects. Thus, mutating T221 does not modify channel activation by canonical Gq-coupled DAG-activation.

### Homologous threonines to T221 in related TRPC3 and TRPC7 confers similar activity control

**Figure 3:**
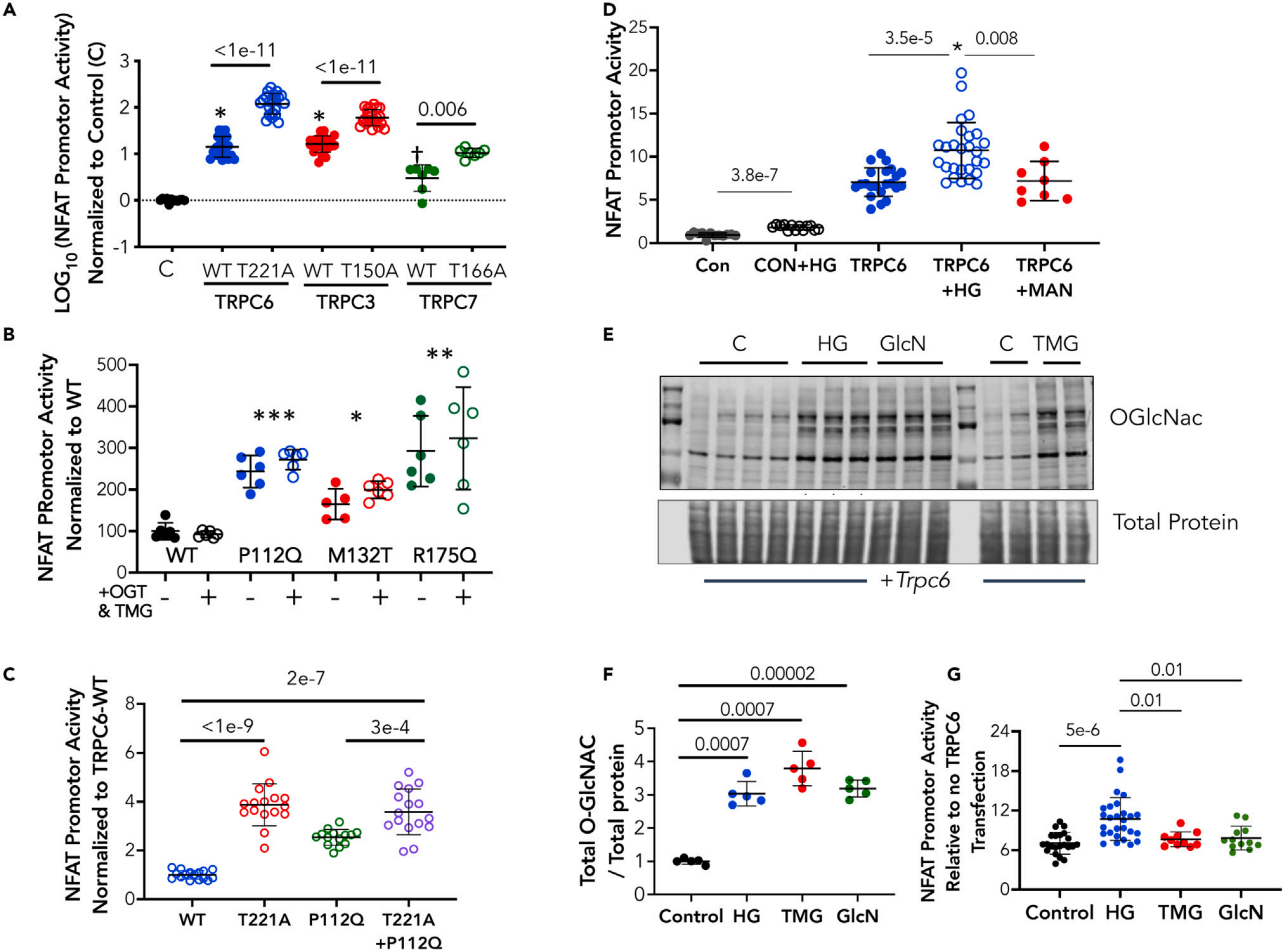
Impact of T→A mutation at homologous residues in TRPC3 and TRPC7 to TRPC6-T221, and role of T221 on TRPC6 gain-of-function mutations or hyperglycemia In all panels showing group data, mean ± SD is displayed for each group. (A) NFAT promoter activity (log-transformed) in control (C, n = 13) and cells expressing either TRPC6 (WT versus T221A); TRPC3 (WT versus T150) (both n = 21) and TRPC7 (WT versus T166) (n = 7). p-values Dunnett’s MCT, Welch ANOVA. (B) NFAT promoter activity in gain of function TRPC6 mutants +/− stimulated O-GlcNAcylation by combined TMG+OGT overexpression. The stimulation did not alter NFAT promoter activity for WT or any mutant. p-values for mutant versus WT: ∗ - 0.033; ∗∗ 0.004; ∗∗∗ 0.0001 – Dunnett’s MCT/Welch ANOVA. (C) NFAT promoter activity in HEK cells expressing T221A, P112Q, or a double mutant TRPC6. The T221A mutation augments NFAT more than P112Q, but their combination further augments to the same level as with T221A alone. 1WANOVA, p-values by Sidak’s MCT. n = 16, 16, 14, 16 for groups, respectively. (D) NFAT promoter activation by hyperglycemia (HG) in HEK-293T cells lacking or expressing TRPC6. Mannitol (Man) serves as negative control. (n = 12–27, p value for-TRPC6 (CON) +/− HG is Mann Whitney U test; p values for +TRPC6 +/− HG or Man are from Kruskal–Wallis test with p-values by Dunn’s MCT. *∗* - p = 0.01 for interaction of HG and TRPC6 expression by 2W-ANOVA. Sample size for each shown at top. (E)Western blot for total O-GlcNAc in lysates from HEK-293T cells treated with 30 mM HG, 10 μM TMG, or 10 μM Glucosamine (GlcN) for 6h. (F) Quantitation of data from experiment E. (n = 5/group, Welch ANOVA, p-values Dunnet’s MCT). (G) Corresponding NFAT promoter activity for the same experimental conditions shown in panel (F). Despite similarly increased levels of O-GlcNAcylation, only high glucose significantly increased NFAT promoter activity over TRPC6-vehicle control. (1WANOVA, p-values by Sidak’s MCT).

Among the seven-member TRPC channel family, TRPC3, TRPC6, and TRPC7 form a closely related subgroup, with TRPC3 and TRPC6 being most homologous.^20^ We therefore tested if the functional role of T221 in TRPC6 was shared by homologous threonines in the other channels. Sequence analysis identified T150 in TRPC3 and T166 in TRPC7 as homologous to T221 in TRPC6 (Figure S3). Each of these were then mutated to alanines, expressed in HEK cells, and the impact on NFAT promoter activation determined (Figure 3A). Each T→A mutation was associated with a near 10-fold rise in NFAT promoter activity over the respective WT channel. These data support conservation of this regulatory region in the structure of all three members of this TRPC subfamily.

### Independence of TRPC6-T221A NFAT activation on GOF mutations or hyperglycemia

Based on our preceding findings, we speculated that gain-of-function TRPC6 mutations that elevate basal NFAT activity may work in part by interfering with T221 O-GlcNAcylation. To test this, vectors expressing several known potent GOF mutations (P112Q, M132T, and R175Q) that amplify resting NFAT promoter activity were studied (Figure 3B). None of these augmented NFAT promoter activity as much as T221A (e.g., 1-2x versus 11x). If the mutations partially interfered with T221 O-GlcNAcylation, we anticipated by mass action that enhancing it with TMG might lower NFAT activity due to the mutation. However, this was not observed with any of the mutations (Figure 3B). Lack of interaction was further tested by expressing T221A, P112Q, and their combination. All three increased NFAT activity, but T221A alone or combined with P112Q achieved the same level (Figure 3C). This supports the conclusion that such GOF mutations unlikely interfere with T221 O-GlcNAcylation as a mechanism for their NFAT activation.

Our initial impetus for exploring O-GlcNAcylation of TRPC6 was triggered a hypothesis that this PTM might be linked to NFAT activation by hyperglycemia (HG). As displayed in Figure 3D, HG stimulates NFAT promotor activation particularly in HEK293 cells expressing TRPC6 (mannitol is used as a negative control). Our results had shown that O-GlcNAcylation reduced NFAT activation, so the notion this was a mechanism of diabetic TRPC6 signaling was refuted. Here we further tested this independence by increasing total protein O-GlcNAcylation by 200–300% with either HG, TMG, or glucosamine, and comparing their impact on NFAT activation. (Figures 3E and 3F). The latter was ony significantly increased by HG despite near identical levels of augmented O-GlcNAcylation by all three interventions (Figure 3G).

### Structural analysis reveals coordinated residues in AK4-linker region that impart basal TRPC6 regulation and O-GlcNAcylation control

**Figure 4:**
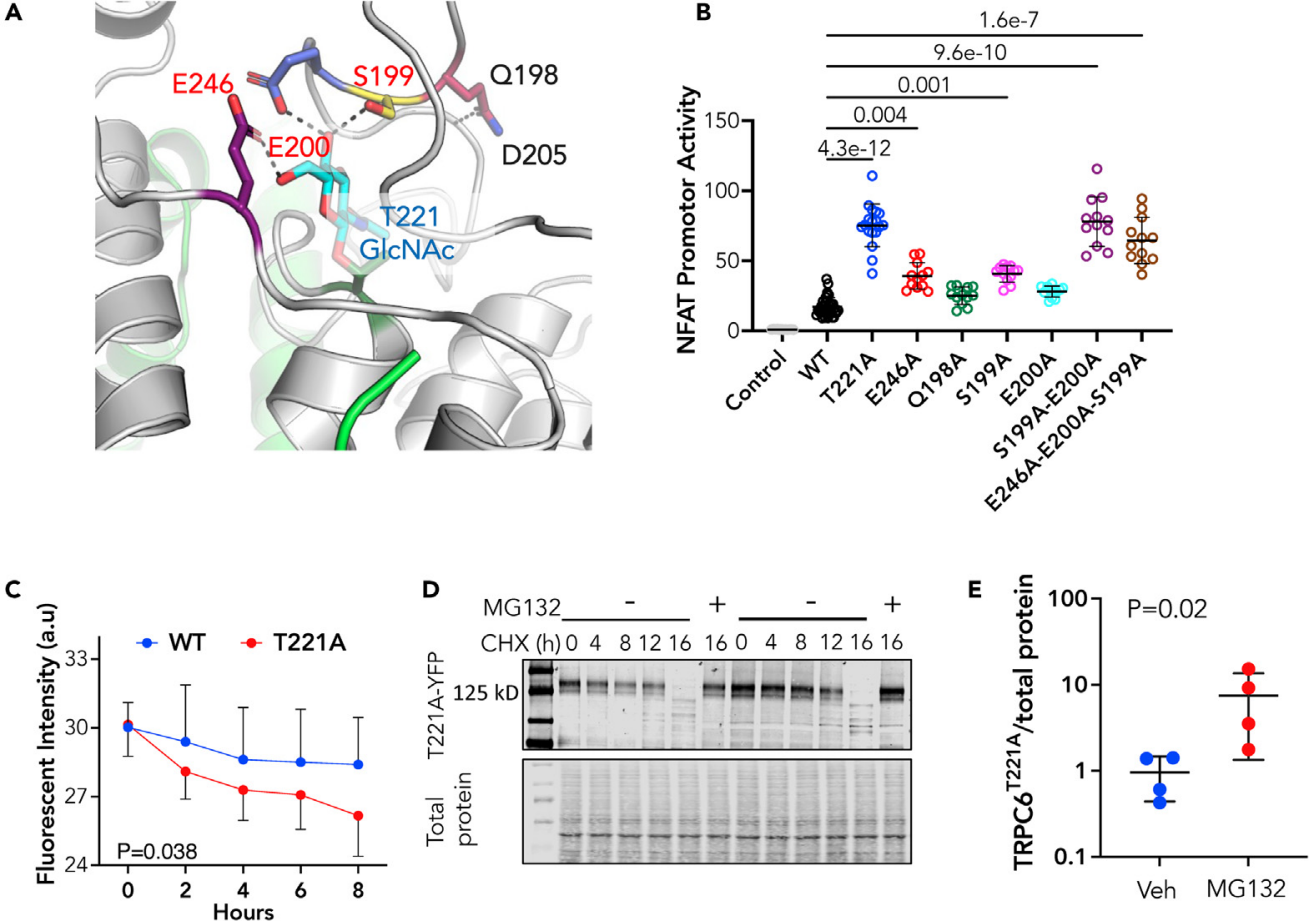
T221-coordinating amino acids in 192-203 Ankyrin Repeat-linker loop region are required for normal constrained TRPC6 channel function In all panels showing group data, mean ± SD is displayed for each group. (A) Structural model of region linking O-GlcNAc modified T221 with AA193-203 loop near ankyrin repeat domain 4 and linker helix. Electrostatic interactions are between E246-*purple*, S199- *yellow*, E200-*blue*. (B) HEK-293T cells transfected with pcDNA plasmid (Control), WT-TRPC6, and TRPC6 mutants impacting these predicted AA interactions. (n = 33 for WT, 8 for T221A, and 12 for all other groups. Kruskal-Wallis test, p-values shown from Dunn’s MCT. (C) Time dependent decline in WT (n = 8) versus T221A (n = 16) protein indexed by decline in YFP fluorescence in cells incubated with cycloheximide (CHX). Mean ± SD; p-value for slope difference by analysis of covariance. (D)Western blot of experiment as depicted in panel (C) with protein expression of TRPC6-YFP shown in cells with or without co-treatment with MG132 to inhibit the proteasome. (E) Densitometry of 16 h after CHX data +/− a proteasome inhibitor (MG 132); (n = 4/group; p value of Mann-Whitney test).

To explore how T221-*O*-GlcNAcylation impacts TRPC6 channel function and identify if other coordinating amino acids are also involved, we turned to recent cryo-electron microscopy structural studies^19^^,^^48^^,^^49^ and modeled interactions influenced by adding an O-GlcNAc modification onto residue T221. The model structure of the relevant region is shown in Figure 4A and predicts T221 O-GlcNAcylation fosters electrostatic contacts between residues within the 193-203 loop in the AR4 domain crossing into the linker helix LH1 domain. The model also predicts O-GlcNAc at T221 enhances electrostatic interaction with glutamic acid (E246, purple) and hydrogen bond formation between the side chain of glutamine (Q198, pink) and the backbone amino group of aspartate (D205, light blue). We posited that these interactions hold a portion of the 193-203 loop in place to enhance S199 and E200 for interaction with O-GlcNAc at T221. The side chains of S199 (yellow), E200 (blue), and E246 are predicted to form hydrogen bonds with the hydroxyl of O-GlcNAc.

To test this, we selectively mutated each residue to an alanine, expressed the recombinant forms of TRPC6 in HEK-293T cells, and assessed NFAT activity. Figure 4B shows results for NFAT activity. E246A and S199A mutations similarly increased NFAT activity over WT, but both less than with the T221A mutation. Neither Q198A nor E200A alone augmented activity over WT, however when both S199A and E200A were combined, the result was comparable with T221A. Adding E246A to form a triple mutant did not further increase NFAT activity. Figure S4 shows an example immunoblot for protein levels obtained with each mutant transfection, with slight changes in some but far less than their impact on corresponding NFAT activity. Thus, the coordinating hydrogen bonds between S199, E200, and O-GlcNAcylated T221 form critical residue interactions regulating basal TRPC6 conductance and NFAT-activation, with E246 itself also impacting this behavior.

### TRPC6 T221 O-GlcNAcylation enhances protein longevity

Beyond stimulating TRPC6 expression, channel conductance and NFAT signaling, we tested if T221 O-GlcNAcylation stabilizes the protein to protect it from proteasomal degradation. T221A or WT TRPC6 were expressed in HEK-293T cells exposed to cycloheximide to block *de novo* protein synthesis +/− the proteasome inhibitor MG132. The rate of protein decline was indexed by GFP fluorescence and was significantly faster with the T221A mutant (Figure 4C). This primarily reflected increased proteasome degradation as MG132 restored levels to baseline similarly with the mutant and WT form (Figures 4D and 4E). Thus, O-GlcNAcylation of TRPC6 reduces its proteasomal degradation to improve post-translational longevity.

## Discussion

In this study, we have identified three amino acids in the AR4-LH1 region of the N-terminus of TRPC6 that are constitutively modified by O-GlcNAc, and find that among these, T221 exerts the dominant control over basal channel current and associated NFAT activation. Preventing O-GlcNAcylation at this residue using a T221A mutation results to our knowledge in the highest basal channel conductance and associated NFAT activity yet reported, including from human GOF mutations that cause renal disease or by other activating PTMs. Consistent with its augmentation of NFAT promotor activity, expression of mutant T221A-TRPC6 amplifies cardiomyocyte pathological hypertrophic gene programs while leaving further receptor-operated (Gq-GPCR) stimulation intact. Although the molecular mechanism for T221A GOF remains to be determined, it appears independent of that associated with GOF mutations. We also find O-GlcNAcylation at T221 coordinates with neighboring amino acids required to constrain channel conductance at normal levels, revealing this region as a key regulatory nexus for the channel. Although we had first hypothesized that TRPC6 O-GlcNAcylation might be a mechanism for HG-stimulated TRPC6-dependent NFAT activation, our data refute this by showing that this modification is already present and that reducing not increasing it results in NFAT activation. These results identify a novel regulatory region of the protein that might be leveraged for therapeutics to constrain hyperactive TRPC6 and associated disease.

It is useful to frame our findings in the context of recent cryo-EM structural data regarding TRPC6.^19^^,^^20^^,^^49^ Such studies reveal TRPC6 tetramers in a two-layered architecture assembled into an inverted bell-shaped intracellular cytosolic domain (ICD) that caps below the transmembrane domain (TMD). The ICD is assembled through interactions between the four ankyrin repeat domain (residues: 96–243) in the N-terminus, linker helices (residues: 256–393) and a coiled-coil domain in the C-terminus. ARs and LHs are key to inter-subunit interactions and TRPC6 tetramer assembly. Specifically, amino acids of the N-terminal loop (85–94) interact with LHs of the neighboring subunit and the last three LHs pack against the TRP helix providing the major contact site between the ICD and TMD. The ARs are highly conserved helix-turn-helix structural motifs^50^^,^^51^^,^^52^ involved in the assembly and stability of multiprotein complexes by forming both intra- and inter-repeat hydrophobic and hydrogen bond interactions.^53^^,^^54^ We find O-GlcNAcylated T221 forms stabilizing electrostatic contacts between AR4, the 193-203 loop near AR4, and loop connecting AR4 to LH1. We believe this helps hold distant regions together and is critical to stabilize the closed state of the channel pore. Many GOF mutations causing renal disease reside in ARs: G109S (AR1), P112Q (AR1), N125S (AR1), M132T (AR2), N143S (AR2), R175Q(AR3), whereas others are found in the C-terminus (e.g. Q899K, R895C, E897K) that is structurally proximate.^20^^,^^48^ Structural studies of these mutants similarly support destabilization of electrostatic interactions at the interface of AR domains and the linker helix that in turn associates with greater current.^49^ A unifying impact of these mutations appears to be suppression of allosteric inhibition by intracellular calcium.^21^ Although our data indicate some of these mutations do not prevent T221 O-GlcNAcylation, it remains possible others may, though this seems unlikely or their impact would be so large as to be embryonically lethal. Indeed, TRPC6 T221 mutations have not been reported in humans. Regardless, identification of the residue cluster T221-*O*-GlcNAc, S199, E200, and E246 and Q198 supports it being a key conserved region required for channel function, linked to constitutive T221 O-GlcNAcylation.

Although attention to O-GlcNAc modifications has often been on its role in disease, it is also known to play constitutive roles.^41^^,^^55^^,^^56^ It is among the most abundant forms of protein glycosylation,^57^ with OGT found in all metazoans and expressed in all mammalian tissues.^58^^,^^59^ OGT also has non-catalytic functionality, but only the O-GlcNAcylation function of OGT appears required for cell survival.^60^ OGT strongly associates with the ribosome and nearly half of ribosomal proteins are O-GlcNAcylated.^61^ For example, O-GlcNAcylation of Sp1 and Nup62 occurs co-translationally and is key to their stability,^62^ whereas that of nuclear pore proteins is required for their functionality.^63^ O-GlcNAcylation is also used to modulate function, as in the case of phospholamban at Ser16 to inhibit myocyte Ca^2+^ uptake by the sarcoplasmic reticulum,^64^ STIM1 where it impedes store-operated Ca^2+^ entry that could influence mechanical and receptor-coupled signaling,^65^ and calcium-calmodulin stimulated kinase II at Ser279 that activates the enzyme.^66^ Although we did not find evidence that further enhancement of constitutive TRPC6 O-GlcNAcylation has biological effects, its reduction caused channel activation. Of the reported post-translational modifiers of TRPC6, only N-glycosylation and from the current results O-GlcNAcylation appear required for normal channel localization and conductance.

Although the current results remove TRPC6 O-GlcNAcylation as a mechanism for its increased activity in hyperglycemic conditions, they do provide a strong example of where O-GlcNAc modifications are required for normal protein function. The data also specifically reveals a previously unknown yet highly conserved regulatory region, sharing homology with closely related channels TRPC3 and TRPC7, and this new insight may help provide new pathways for therapeutics to modify channel function.

### Limitations of the study

Although site mutagenesis is commonly used to study the functional impact of targeted post-translational protein modifications, and alanine substitutions will prevent O-GlcNacylation at a given site, it has limitations. Specifically, one may observe changes that are because of substituting the native amino acid to alter protein structure/function itself. Although we cannot fully rule out this possibility we believe the data support a primary effect related to prevention of O-GlcNAcylation. First, we find reducing O-GlcNAcylation with OGT inhibition also increases O-GlcNAcylation of TRPC6 and NFAT activation, yet has significantly less impact on either behavior when the T221A mutation is expressed. Second, the modeling analysis predicts local residue interactions based on T221A being O-GlcNAcylated and not just mutated to alanine, and these predictions are experimentally confirmed. The molecular weights for TRPC6-YFP in our IP gel studies are close to predicted, but did differ somewhat from gel to gel. In particular, we found the weights of the immunoblot from the IP lysates a bit lower than in the input material. This may reflect loss of some of the PTMs on the protein such as N-glycosylation. Still, finding TRPC6 O-GlcNAcylation by IP assay, mass spectrometry, and click-chemistry assay, and reduced changes in the T221A mutation supports this as directly modifying TRPC6. Lastly, the mechanism by which T221 OGlcNAcylation and its interactants control channel conductance and associated NFAT activity remains to be determined, but maybe identified by a future cryo-electron microscopy analysis.

## STAR★Methods

### Key resources table

**Table:** 

REAGENT or RESOURCE	SOURCE	IDENTIFIER
**Antibodies**		

O-GlcNAc (CTD 110.6) (Mouse Monoclonal antibody)	O-GlcNAc Core, JHU, Ma et al. ^67^	N/A
OGT (Rabbit Polyclonal antibody)	Sigma-Aldrich	Cat# O6264; RRID:AB_532313
GFP (Rabbit Polyclonal antibody)	Thermo Fisher Scientific	Cat# A-6455; RRID:AB_221570
GFP M-trap beads, Chromo Tek GTD-20	Thermo Fisher Scientific	Cat#17373353
IRDye 800CW donkey anti-rabbit 800	LI-COR	Cat#926-32213; RRID:AB_621848
IRDye 800CW donkey anti-mouse 680	LI-COR	Cat#926-32212; RRID:AB_621847
IRDye 800CW goat anti-rabbit 800	LI-COR	Cat#926-32211; RRID:AB_621843
IRDye 800CW goat anti-mouse 680	LI-COR	Cat#926-32210; RRID:AB_621842
IRDye 800CW Streptavidin dye	LI-COR	Cat#926-32230

**Bacterial and virus strains**		

pAV-YFP adenovirus	This paper	N/A
pAV-hTRPC6 WT_YFP adenovirus	This paper	N/A
pAV-hTRPC6 T221A_YFP adenovirus	This paper	N/A

**Chemicals, peptides, and recombinant proteins**		

Dulbecco’s modified Eagle’s medium	Thermo Fisher Scientific	Cat#11965084
Fetal bovine serum	Sigma-Aldrich	Cat#F2442
Glucose	Sigma-Aldrich	Cat#G8270
Mannitol	Sigma-Aldrich	Cat#M4125
Thiamet G	O-GlcNAc Core, JHU	N/A
Glucosamine	Sigma-Aldrich	Cat#G4875
PBS	Thermo Fisher Scientific	Cat#10010023
RIPA buffer	Sigma-Aldrich	Cat#R0278
Complete™ protease inhibitor cocktail	Roche Diagnostics	Cat#11836153001
Trizol Reagent	Thermo Fisher Scientific	Cat#15596026
PNGase F	New England Biolabs	Cat#P0704S
PierceTM Protein A/G Magnetic Beads	Thermo Fisher Scientific	Cat#88802
4–15% Criterion™ TGX Stain-Free™ Protein Gels	Bio-Rad Laboratories	Cat#5678085
Trans-Blot® Turbo™ Midi Nitrocellulose Transfer Packs	Bio-Rad Laboratories	Cat#1704159EDU
Cycloheximide	Sigma-Aldrich	Cat#C7698
MG132	Sigma-Aldrich	Cat#M8699
Osmi1	Sigma-Aldrich	Cat#SML1621

**Critical commercial assays**		

GeneArt Site-Directed Mutagenesis System	Thermo Fisher Scientific	Cat# A13282
Xfect transfection reagent	Takara Bio	Cat#631318
Dual Luciferase Reporter Assay Kit	Promega	Cat#E1910
Click-iT GlcNAz metabolic glycoprotein labeling reagents	Thermo Fisher Scientific	Cat#C33368
	Thermo Fisher Scientific	Cat#C33372
High-Capacity RNA-to-cDNA Kit	Applied Biosystems, Thermo fisher	Cat#4388950

**Experimental models: Cell lines**		

HEK-293T	ATCC	ATTC: CRL-3216
Neonatal Rat cardiomyocytes (NRVMs)	Primary cultured	N/A

**Oligonucleotides**		

*Trpc6*	Thermo Fisher Scientific	Assay ID# Rn00677559_m1
*Nppa*	Thermo Fisher Scientific	Assay ID# Rn00664637_g1
*Nppb*	Thermo Fisher Scientific	Assay ID# Rn00580641_m1
*Myh7*	Thermo Fisher Scientific	Assay ID# Rn01488777_g1
*Rcan1*	Thermo Fisher Scientific	Assay ID# Rn01458494_m1
*Gapdh*	Thermo Fisher Scientific	Assay ID# Rn01775763_g1

**Recombinant DNA**		

pcDNA3-human TRPC6-YFP	Koitabashi et al.^30^	N/A
pcDNA3- YFP	This paper	N/A
pcDNA3- human TRPC3	Seo et al.^3^	
pcDNA3- human TRPC7	Dr. Steve S Pullen (Boehringer Ingelheim)	N/A
FLAG-OGT	Dr. Gerald W Hart, O-GlcNAc Core, JHU	N/A
pGL3 Luc	Promega	E1761
pGL4.30-NFAT-RE-luc	Seo et al.^3^	N/A
pGL4.74-TK (thymidine kinase)	Seo et al.^3^	N/A
TRPC3-YFP T150A	This paper	N/A
TRPC7-YFP T166A	This paper	N/A
TRPC6-YFP S14A	This paper	N/A
TRPC6-YFP T70A	Koitabashi et al.^30^	N/A
TRPC6-YFP T221A	This paper	N/A
TRPC6-YFP E246A	This paper	N/A
TRPC6-YFP Q198A	This paper	N/A
TRPC6-YFP S199A	This paper	N/A
TRPC6-YFP E200A	This paper	N/A
TRPC6-YFP S14AT221A	This paper	N/A
TRPC6-YFP S14AT70AT221A	This paper	N/A
TRPC6-YFP S199AE200A	This paper	N/A
TRPC6-YFP E246AS199AE200A	This paper	N/A
TRPC6 gene promoter construct	This paper	N/A
TRPC6-YFP P112Q	This paper	N/A
TRPC6-YFP P112QT221A	This paper	N/A
TRPC6-YFP M132T	This paper	N/A
TRPC6-YFP R175Q	This paper	N/A

**Software and algorithms**		

Analyst TF 1.7 software	Aldeghaither et al.^68^	N/A
Protein Pilot version 5.0 software	Aldeghaither et al.^68^	N/A
Paragon and Progroup algortihms	Aldeghaither et al.^68^	N/A
Prism Ver 9.3.1	GraphPad.com	N/A

### Resource availability

#### Lead contact

All requests for reagents and resources should be directed to the lead contact, David A Kass (dkass@jhmi.edu).

#### Materials availability

Plasmids generated in this study are available on reasonable request to the lead contact.

### Experimental model and subject details

#### Cell line

The HEK-293T (ATTC: CRL-3216, (RRID: CVCL_0063) cell line was utilized in the in vitro assays. HEK-293T cells were grown in Dulbecco’s modified Eagle’s medium supplemented with 10% fetal bovine serum, 2 mM glutamine, 1 mM pyruvate and antibiotics.

#### Primary cell culture

Some experiments were conducted in primary cultured neonatal rat ventricular myocytes as mentioned in the methods sections. Cells were cultured in DMEM with 10% FBS and 1% penicillin/streptomycin.

### Method details

#### Plasmids

pcDNA3-human TRPC6-YFP plasmid was obtained from Dr. Craig Montell (Koitabashi et al., 2010;^30^ Kwon et al., 2007^69^), pcDNA3-human TRPC3 plasmid from Dr. Jeffery Molkentin (Seo et al., 2014b),^5^ pcDNA3-human TRPC7 from Dr. Steve S Pullen (Boehringer Ingelheim) and FLAG-OGT from Dr. Gerald W Hart. pGL4.30-NFAT-RE firefly luciferase (NFAT-luc) driven by the NFAT response element and Renilla luciferase (TK-Rluc) vectors were from Promega (Seo et al., 2014b).^5^ Alanine substitution mutants: TRPC6-YFP: S14A, T70A, T221A, S14AT221A, S14AT70AT221A, E246A, Q198A, S199A, E200A, S199AE200A, E246AS199AE200A, TRPC3-YFP: T150A; Gain-of-function TRPC6 mutants (P112Q, M132T, R175Q), P112QT221A; TRPC3-YFP: T150A;and TRPC7-YFP: T166A were each generated by PCR-based site mutagenesis (GeneArt Site-Directed Mutagenesis System, A13282) using pcDNA3-human TRPC6-YFP, TRPC3-YFP and TRPC7-YFP as the template. A human TRPC6 gene promoter construct was made by cloning a 1.7kb insert corresponding to the upstream of transcription start site of Human TRPC6 into luciferase reporter plasmid (pGL3 Luc, Promega E1761). pcDNA3 and pcDNA3-YFP vectors served as the control for transfection assays.

#### HEK-293T cell transfection and luciferase promotor assay

HEK-293T cells were grown in Dulbecco’s modified Eagle’s medium supplemented with 10% fetal bovine serum, 2 mM glutamine, 1 mM pyruvate and antibiotics. For each well of a 48 well plate, cells were cultured to 70% confluence and transfected with plasmids encoding NFAT-luc (0.25 μg), TK-Rluc (0.02 μg internal control) and wildtype or alanine substituted TRPC mutants (0.25 μg). Transfection was carried out using Xfect transfection reagent following manufacturer’s instruction (Takara Bio, 631318). After transfection, cultures were maintained in serum containing medium for 24 h. After 24h, all treatments were done in serum free culture medium. For High glucose (HG) exposure experiments, DMEM supplemented with 30 mM glucose (Sigma-Aldrich G8270) or 30 mM mannitol (Sigma-Aldrich M4125) (as HG control) was used. OGA inhibitor Thiamet G (TMG) (O-GlcNAc Core, JHU) was used at a concentration of 10 μM and glucosamine (GlcN) (Sigma-Aldrich G4875, 2 mM) was treated for 6h to stimulate O-GlcNAcylation (Chatham and Marchase, 2010^70^). Cells were harvested using passive lysis buffer (Promega E1910). NFAT-luciferase activity was determined using Dual Luciferase Reporter Assay Kit (Promega E1910) using the manufacturer protocol.

#### Immunoprecipitation assay

HEK- 293T cells were plated in 10 cm cell culture dishes and grown to 70% confluency. Cells were transfected with 10μg of OGT-Flag and TRPC6-YFP plasmids using Xfect transfection reagent following manufacturer’s instruction (Takara Bio, 631318) and maintained in serum containing medium for 24 h to express the respective proteins. In some studies, cells were further treated with Thiamet G (10 μM) to stimulate O-GlcNAcylation for 24h before harvesting. Cells were washed in ice-cold PBS (ThermoFisher Scientific 10010023), resuspended in RIPA buffer (Sigma-Aldrich R0278) containing Complete™ protease inhibitor cocktail (Roche Diagnostics, 11836153001) and incubated for 20 min on ice to complete lysis. TMG (10 μM) was also added to the lysis buffer in cells pre-treated with TMG. Cell lysates were centrifuged at 12000 rpm × 10minat 4°C and supernatants were used for immuno-precipitation (IP) analysis. In some studies, PNGase F digestion (PNGase F, New England Biolabs P0704S) was done per manufacturer’s specifications to remove N-linked glycans prior to IP studies as indicated in the results section. IP was carried out by incubating lysates with OGT antibody (Sigma-Aldrich O6264), OGlcNAc antibody (CTD 110.6, MABS1254), or GFP M-trap beads (Chromotek GTD-20 serves as YFP capture beads). For OGT and OGlcNAc IP reactions, 50 μL slurry of PierceTM Protein A/G Magnetic Beads (ThermoFisher Scientific 88802) were added to 200 μL of OGT and OGlcNAc IP lysates and all IP samples were incubated for 4h at 4°C with gentle rolling. GFP M-trap beads were directly used for TRPC6-YFP pull down. GFP M-trap beads and OGT IP magnetic beads were washed thrice with 1 mL of washing buffer (20 mM Tris/HCl, pH 7.4, 150 mM NaCl, 1 mM EDTA, 0.05% Triton X100, 5% glycerol and Complete™ protease inhibitor cocktail) and proteins were eluted in 30 μL SDS-PAGE loading buffer. O-GlcNAc IP magnetic beads were washed 3x in TBS and eluted with 15 μL of 1M GlcNAc in TBS. Enriched proteins and 10% input samples were boiled in 30 μL of 2× SDS-PAGE loading buffer containing 5% 2-mercaptoethanol for 5 min, and further used for SDS-PAGE analysis.

#### SDS/PAGE and western blot analysis

Key resources table contains information of primary and secondary antibodies used in this study. For Western blot analysis, protein samples were separated by precast 4–15% Criterion™ TGX Stain-Free™ Protein Gels (Bio-Rad Laboratories 5678085), transferred to a nitrocellulose membrane using Trans-Blot® Turbo™ Midi Nitrocellulose Transfer Packs (Bio-Rad Laboratories 1704159EDU) and Trans-Blot® Turbo™ Transfer System (Bio-Rad Laboratories 1704150). The membranes were blocked with 5% non-fat dried skim milk solution for 1 hat 27°C, then incubated overnight at 4°C with primary antibodies diluted in blocking buffer: anti-OGT(1:10,000), anti-O-GlcNAc(1:10,000) and anti-GFP (1:10,000). The next day, fluorescent secondary antibodies (IRDye 800CW donkey anti-rabbit 800(1:20,000), IRDye 800CW donkey anti-mouse 680(1:20,000), IRDye 800CW goat anti-rabbit 800 (1:20,000) and IRDye 800CW goat anti-mouse 680 (1:20,000)), diluted in 1% milk in TBS-T, were added to the membranes for 1 hat room temperature. The membranes were washed 3x in TBS-T for 10 min each and subsequently images were acquired and analyzed using the LI-COR Odyssey Image System.

#### O-GlcNAc Click-iT GlcNAz metabolic labeling of WT-TRPC6

O-GlcNAcylation of TRPC6 was assayed by Click-iT GlcNAz metabolic glycoprotein labeling reagents (ThermoFisher Scientific C33368). Lysates (200 μg protein) from HEK-293T cells expressing WT-TRPC6-YFP was incubated with 50 μL GFP M-trap beads (Chromotek GTD-20) to immunoprecipitate TRPC6. Immunoprecipitated protein was enzymatically labeled utilizing the permissive mutant β-1,4-galactosyltransferase (Gal-T1 Y289L) which transfers azido-modified galactose (GalNAz) from UDP-GalNAz to O-GlcNAc residues on the target proteins as per manufacturer’s specifications (ThermoFisher Scientific C33368). The labelled lysate was then clicked on with biotin-alkyne using copper catalyzed azide-alkyne click chemistry reaction protocol according to manufacturer’s instruction (ThermoFisher Scientific C33372). The biotinylation was detected using IRDye 800CW Streptavidin dye and imaged (Odyssey Image System LICOR).

#### nanoACQUITY UltraPerformance LC mass spectrometry

pcDNA3-human TRPC6-YFP was over-expressed in HEK-293T cells and immunoprecipitated. The eluate was subjected to SDS-PAGE followed by Commassie Blue staining. The corresponding gel bands were cut out and excised into cubes (ca. 1 × 1 mm) with a razor blade. Gel pieces were de-stained with 50% ACN followed by the addition of 100 μl of 10 mM dithiothreitol (DTT) in 50 mM bicarbonate buffer and incubation at 37°C for 0.5 h. After removal of DTT solution, 100 μL of 30 mM iodoacetamide in 50 mM bicarbonate buffer was added and incubated in dark for 30 min. Proteins were then digested with the addition of sequencing-grade trypsin/Lys-C followed by incubation at 37°C overnight. The yielded peptides were extracted and desalted with C18 Ziptip columns, with elutes dried down with a SpeedVac. Extracted peptides were analyzed with a NanoUPLC-MS/MS system integrating nanoAcquity UPLC (Waters) and a TripleTOF 6600 mass spectrometr (Sciex) (by using similar settings shown in a previous report (Aldeghaither et al., 2019), with some modifications. Specifically, dried peptides were dissolved in 0.1% formic acid and loaded onto a C18 Trap column (Waters Acquity UPLC Symmetry C18 NanoAcquity 10 K 2G V/M, 100 A, 5 μm, 180 μm × 20 mm) at 15 μL/min for 2 min. Peptides were then separated with an analytical column (Waters Acquity UPLC M-Class, peptide BEH C18 column, 300 A, 1.7 μm, 75 μm × 150 mm) which was temperature controlled at 40°C. The flow rate was set as 400 nL/min. A 60-min gradient of buffer A (2% ACN, 0.1% formic acid) and buffer B (0.1% formic acid in ACN) was used for separation: 1% buffer B at 0 min, 5% buffer B at 1 min, 45% buffer B at 35 min, 99% buffer B at 37min, 99% buffer B at 40 min, 1% buffer B at 40.1 min, and 1% buffer B at 60 min. Data were acquired with the TripleTOF 6600 mass spectrometer using an ion spray voltage of 2.3kV, GS1 5 psi, GS2 0, CUR 30 psi and an interface heater temperature of 150°C. Mass spectra was recorded with Analyst TF 1.7 software in the IDA mode. Each cycle consisted of a full scan (m/z 400-1600) and fifty information dependent acquisitions (IDAs) (m/z 100-1800) in the high sensitivity mode with a 2+ to 5+ charge state. Rolling collision energy was used.

Data files were submitted for simultaneous searches using Protein Pilot version 5.0 software (Sciex) utilizing the Paragon and Progroup algortihms and the integrated false discovery rate (FDR) analysis function. MS/MS data was searched against the customized human TRPC6 protein database. Trypsin/LysC was selected as the enzyme. Carbamidomethylation was set as a fixed modification on cysteine. HexNAc emphasis was chosen as a special factor. Other search parameters include instrument (TripleTOF 6600), ID Focus (Biological modifications), search effort (Thorough), false discovery rate (FDR) analysis (Yes), and user modified parameter files (No). The proteins were inferred based on the ProGroupTM algorithm using ProteinPilot software. The detected protein threshold in the software was set to the value which corresponded to 5% FDR. Peptides were defined as redundant if they had identical cleavage site(s), amino acid sequence, and modification. All peptides were filtered with confidence to 5% FDR, with the confidence of HexNAc sites automatically calculated. Each of the HexNAc modification sites (>95% confidence) was then manually confirmed and annotated (Ma and Hart, 2017).^67^

#### Electrophysiology studies – Patch clamp

Patch clamp studies were done as previously described protocol (Galvis-Pareja et al., 2014^71^). Briefly, HEK-293T cells were plated in glass coverslips in a 24 well plate and transfected with 1 μg of TRPC6 channel plasmids (WT, T221A and P112Q) using Xfect transfection reagent according to manufacturer’s protocol (Takara Bio 631318). Cells expressing wildtype and mutant channels were identified by YFP fluorescence. The bath solution was 140mM NaCl, 5mM CsCl2, 1 mM MgCl2, 10 mM HEPES, and 10 mM glucose with pH of 7.4. Borosilicate glass capillary pipettes (World Precision Instr.) were used with ∼3MΩ resistance when filled with solution containing 5mM NaCl, 40 mM CsCl2, 80mM Cs-glutamate, 5mM Mg-ATP, 5 mM EGTA, 1.5 CaCl2 (free calcium concentration was 100 nM). Currents were obtained using a voltage step-pulse protocol or ramp protocol from -100 mV to +100 mV applied every 2s for 500 ms from holding potential of -60 mV. Current recordings were in a whole-cell configuration using Axopatch 200A amplifier (Axon Instruments, Molecular Devices).

#### Neonatal Rat ventricular myocyte isolation and adenoviral transfection

Neonatal Rat cardiomyocytes (NRVMs) were freshly isolated as previously described (Mishra et al., 2021^72^) and cultured at 1 million cells per well in six-well plates for 24 h in DMEM with 10% FBS and 1% penicillin/streptomycin. Adenoviruses were developed expressing the GFP-tagged wild-type sequence of human TRPC6 or TRPC6 T221A. NRVMs were infected with an MOI of 10 with the respective viruses for 48 h before performing the downstream assays.

#### RNA isolation and gene expression analysis

Total RNA from NRVMs was extracted using Trizol Reagent (Cat. No. 15596026, Invitrogen, Thermofisher, USA) per manufacturer’s instructions. High-Capacity RNA-to-cDNA Kit (Cat. No. 4388950, Applied Biosystems, Thermofisher, USA) was used to reverse transcribe the RNA into cDNA as described before (Mishra et al., 2021^72^). Quantitative real time PCR analysis was carried out using TaqMan specific primers for: Trpc6, Nppa, Nppb, Myh7, Rcan1, and Gapdh. Primer information are displayed in the key resources table. The threshold cycle (Ct) values were determined by crossing point method and normalized to GAPDH (Applied Biosystems) values for each run.

#### Protein degradation measurement

A fluorescent microplate-based assay was used to measure TRPC6-WT versus TRPC6-T221A-GFP signal intensity decay. HEK-293T cells seeded on 96 well plates were transfected with 0.1 μg of the respective plasmids. 24 h after transfection, cells were treated with cycloheximide (100 μg/mL), and fluorescence intensities of the wells were measured at the indicated time points. T221A-GFP decline was measured in cycloheximide treated cells in the presence or absence of proteasome inhibitor MG132 (10 μM). For western blot experiments, cells were plated in 6 well dishes and transfected with 5μg plasmids per well. 24 h after transfection, HEK cells were incubated with 100 μg/mL of cycloheximide. Cells were harvested using100 μL RIPA cell lysis buffer at the indicated time points as shown in Figure 4D, followed by SDS-PAGE and western blotting to visualize TRPC6 T221A protein levels.

#### Statistical analysis

Statistical analysis was performed using Prism Ver 9.3.1. All of the individual tests used for each datafigure in the study are provided in their respective figure legend along with sample size per group. For multiple groups, 1-way ANOVA, a Welch ANOVA (if test for variance difference between groups was positive), or non-parametric Kruskal Wallis (if non-normally distributed) was used. A 2-Way ANOVA was also used in some testing as indicated. Two-group comparisons used non-parametric tests (Mann Whitney U test). All precise p values are provided for statistical testing in the figures and/or legends.

## Data Availability

All data reported in this paper will be shared by the lead contact upon request. This paper does not report original code.
