# Supplementary material for: Transient receptor potential canonical type 6 (TRPC6) O-GlcNAcylation at Threonine-221 plays potent role in channel regulation
Source: iScience. 2023 Feb 28;26(3):106294. doi: 10.1016/j.isci.2023.106294 (PMC10014292; doi:10.1016/j.isci.2023.106294)

iScience, Volume *26*

Supplemental information

Transient receptor potential canonical type 6 (TRPC6) O-GlcNAcylation at Threonine-221 plays potent role in channel regulation

Sumita Mishra, Junfeng Ma, Desirae McKoy, Masayuki Sasaki, Federica Farinelli, Richard C. Page, Mark J. Ranek, Natasha Zachara, and David A. Kass

Supplemental Figure S1:

TRPC6 double band reflects N-glycosylated upper band, and ~125 Kd lower band. Example immunoblot of lysates from HEK293 expressing TRPC6-YFP that were then incubated with PNGase F to remove N-glycosylation or with vehicle. The usual higher MW band of TRPC6 is N-glycosylated, and its removal shifts the weight down closer to 125 kD as known to occur. Related to Figure 1D.

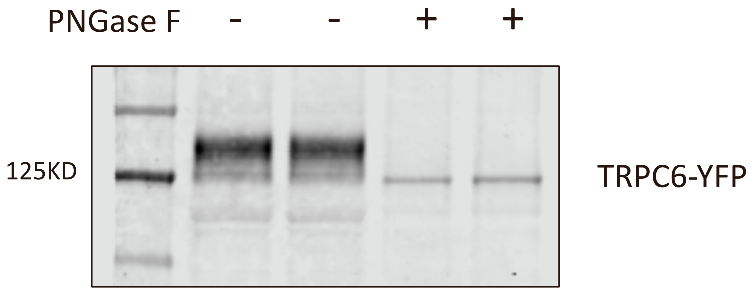

Supplemental Figure S2:

Threonine 221 in TRPC6 is highly conserved among species.

Amino acid sequence in AK4-LK1 region of TRPC6 where T221 and coordinating amino acids reside is highly conserved across many species. Related to Figure 1G, 1H.

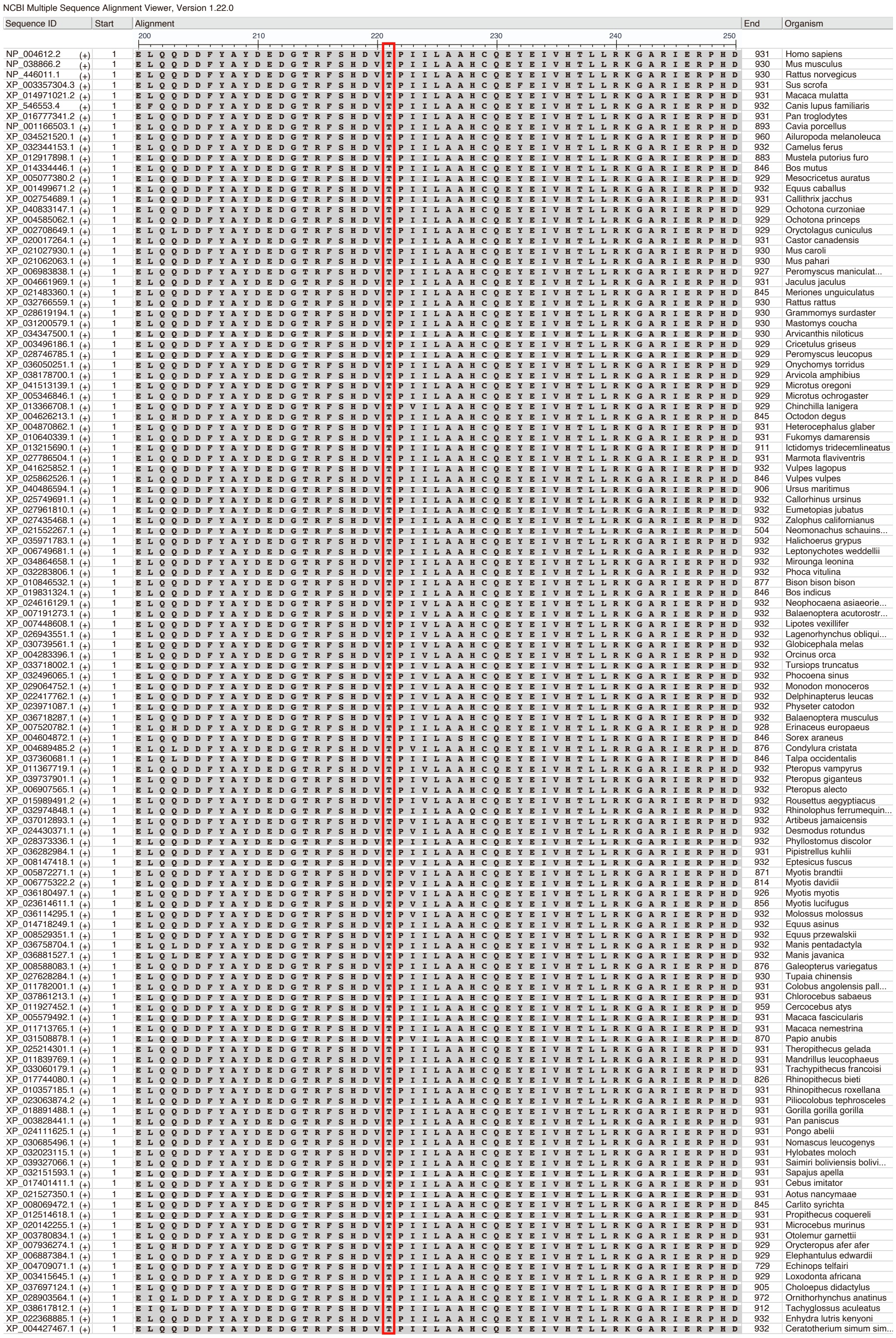

Supplemental Figure S3:

Identification of homologous threonine to TRPC6 T221 in closely related TRPC channels TRPC3, and TRPC7. Amino acid protein sequence alignment of all three channels are displayed and threonine T221 in TRPC6 corresponds to T150 in TRPC3 and to T166 in TRPC7. All are surrounded by highly conserved sequences. Related to Figure 3A.

```
sp|Q9Y210|TRPC6_HUMAN      DNRLAHRRQTVLREKGRRLANRGPAYMFSDRSTSLSIEEERFLDAAEYGNIPVVRKMLEE 120
sp|Q13507|TRPC3_HUMAN      -----------MREKGRRQAVRGPAFMFNDRGTSLTAEEERFLDAAEYGNIPVVRKMLEE 49
sp|Q9HCX4|TRPC7_HUMAN      TFKNMQRRHTTLREKGRRQAIRGPAYMFNEKGTSLTPEEERFLDSAEYGNIPVVRKMLEE 65
                                      :****** * ****:**.::.***: *******:***************

sp|Q9Y210|TRPC6_HUMAN      CHSLNVNCVDYMGQNALQLAVANEHLEITELLLKKENLSRVGDALLLAISKGYVRIVEAI 180
sp|Q13507|TRPC3_HUMAN      SKTLNVNCVDYMGQNALQLAVGNEHLEVTELLLKKENLARIGDALLLAISKGYVRIVEAI 109
sp|Q9HCX4|TRPC7_HUMAN      SKTLNFNCVDYMGQNALQLAVGNEHLEVTELLLKKENLARVGDALLLAISKGYVRIVEAI 125
                           .::**.***************.*****:**********:*:*******************

sp|Q9Y210|TRPC6_HUMAN      LSHPAFAEGKRLATSPSQSELQQDDFYAYDEDGTRFSHDVTPIILAAHCQEYEIVHTLLR 240
sp|Q13507|TRPC3_HUMAN      LNHPGFAASKRLTLSPCEQELQDDDFYAYDEDGTRFSPDITPIILAAHCQKYEVVHMLLM 169
sp|Q9HCX4|TRPC7_HUMAN      LNHPAFAQGQRLTLSPLEQELRDDDFYAYDEDGTRFSHDITPIILAAHCQEYEIVHILLL 185
                           *.**.** .:**: ** :.**::************** *:**********:**:** **

sp|Q9Y210|TRPC6_HUMAN      KGARIERPHDYFCKCNDCNQKQKHDSFSHSRSRINAYKGLASPAYLSLSSEDPVMTALEL 300
sp|Q13507|TRPC3_HUMAN      KGARIERPHDYFCKCGDCMEKQRHDSFSHSRSRINAYKGLASPAYLSLSSEDPVLTALEL 229
sp|Q9HCX4|TRPC7_HUMAN      KGARIERPHDYFCKCNECTEKQRKDSFSHSRSRMNAYKGLASAAYLSLSSEDPVLTALEL 245
                           ***************.:* :**::*********:******** ***********:*****

sp|Q9Y210|TRPC6_HUMAN      SNELAVLANIEKEFKNDYKKLSMQCKDFVVGLLDLCRNTEEVEAILNGDVETLQ--SGDH 358
sp|Q13507|TRPC3_HUMAN      SNELAKLANIEKEFKNDYRKLSMQCKDFVVGVLDLCRDSEEVEAILNGDLESAEPLEVHR 289
sp|Q9HCX4|TRPC7_HUMAN      SNELARLANIETEFKNDYRKLSMQCKDFVVGVLDLCRDTEEVEAILNGDVNFQV--WSDH 303
                           ***** *****.******:***********:*****::**********::       .:
```

Supplemental Figure S4:

Equal levels of protein expressed in HEK cells by plasmids encoding various gain of function TRPC6 mutations. HEK cells were transfected with mutations targeting nearby serine (S), glutamine (Q), and glutamic acid (E) residues in TRPC6 that were predicted by molecular modeling to interact with threonine 221 to control resting channel conductance and associated NFAT activation. Each mutation converted the original amino acid to an alanine. For those with multiple mutations, they were encoded in a single plasmid. Related to Figure 4B.

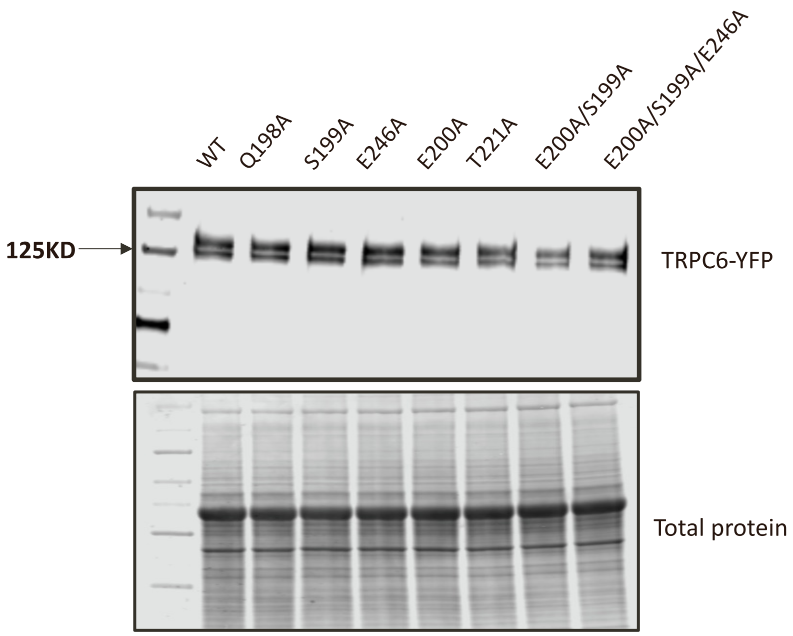

Supplement: Document S1. Figures S1–S4 [file mmc1.pdf]
